# Future prospects for prophylactic and therapeutic management of venous thrombosis: antithrombotic substances with lower risk of hemorrhage?

**DOI:** 10.1590/1677-5449.190036

**Published:** 2019-05-28

**Authors:** Francisco Humberto de Abreu Maffei

**Affiliations:** 1 Universidade Estadual Paulista – UNESP, Faculdade de Medicina de Botucatu, Departamento de Cirurgia e Ortopedia, Botucatu, SP, Brasil.

Anticoagulants are medications that have been essential for treatment and prophylaxis of venous thromboembolism since the 1940s, initially with heparins and vitamin K antagonists. However, even with the introduction of low molecular weight heparins, fondaparinux, and, more recently, direct oral anticoagulants, there is still a non-negligible risk of significant and even lethal hemorrhages.^1^^-^ 4

Knowledge that has been accumulated over recent decades, primarily from experimental studies, suggests that the immune system and inflammatory cells play a role in initial and localized activation of coagulation in the veins, triggering thrombosis, primarily in situations in which blood flow is reduced or blocked.^5^^-^ 8 This state of stasis can occur in human beings in cases of venous compression and restriction to bed because of clinical diseases or surgery and also during anesthesia, immobilization due to trauma, paralysis, and long journeys.^9^^-^ 11

Studies in experimental models of thrombosis, provoked by reducing or halting blood flow by induced stenosis or ligature in the vena cava of rodents suggest that, in response to ischemia and activation of endothelial cells, molecules are released that attract leukocytes and platelets and adhesion molecules for these cells are also exposed in the endothelium. It has also been demonstrated that the leukocytes that adhere are primarily monocytes that release tissue factor (TF) and neutrophils that release enzymes and form neutrophil extracellular traps (NETs), which activate coagulation factors and deactivate natural anticoagulants.^12^^-^ 14 Von Brühn et al.^15^ have shown in a highly illustrative manner, using scanning electron microscopy, that induction of stenosis in the vena cava of mice does not provoke morphological injury to the endothelium. However, they observed that after 1 hour leukocytes began to roll along the endothelium and after 6 hours the surface of the endothelium was covered by a layer of these cells. Using intravital microscopy, they showed that these leukocytes were primarily monocytes and neutrophils. They also showed presence of NETs in thrombi and their role in formation and progression of the thrombus by activation of the intrinsic coagulation system. To achieve this, they used transgenic animals with neutropenia or with factor XII deficiency and animals in which NETs were lysed by DNase, in which formation of thrombus would not occur.

The following question therefore arises: could it be possible to use substances with anti-inflammatory activity that inhibits these mechanisms and with weaker or nonexistent systemic anticoagulant effects to treat venous thrombosis (VT)?

Based on the knowledge described above, a number of different substances have been used with the objective of inhibiting molecules responsible for attraction or adhesion of inflammatory cells, such as P-selectins and E-selectins, or of inhibiting enzymes released by these cells, which locally activate the coagulation system or act at some point in this initial sequence of events involved in thrombi development, in the hope of impeding their formation or progression, without interfering with systemic coagulation.

Many different studies published by Dr. Wakefield’s University of Michigan team have demonstrated the antithrombotic activity of P-selectin inhibitors (an adhesion molecule for platelets and leukocytes), both in a model of thrombosis induced by ligature of the vena cava in rats^16^^,^ 17 and in monkeys, inducing thrombosis in the vena cava or iliac veins using balloon occlusion.^18^^-^ 21

Culmer et al.,^22^ part of the same team, using an E-selectin inhibitor for prevention and treatment of thrombosis in a model of vena cava stasis in mice, also demonstrated an inhibitory effect on formation and extension of thrombi in a similar manner to enoxaparin, in relation to a control group, without changing the bleeding time, as occurs with enoxaparin.

In a study along the same lines, we tested substances with anti-inflammatory activity for prevention of VT in the Protein Purification Laboratory, Department of Biochemistry, UNIFESP (Prof. Dr. Maria Luiza Vilela Oliva), using the recombinant inhibitor rBbCI,^23^ the original protein of which has demonstrated inhibitory actions on elastase, cathepsin-G,^24^ proinflammatory enzymes that are also inhibited by heparin.^25^ The rBbCI inhibitor also reduced levels of interleukin-8, a cytokine that primarily stimulates migration of neutrophils to the focus of inflammation.^24^ In a model of vena cava ligature in rats, rBbCI had an inhibitory effect on development of the thrombus similar to that of heparin and, like heparin, the action was dose-dependent.^26^ However, rBbCI did not change activated partial thromboplastin time or bleeding time in the animals’ tails, which were identical to times in control animals. The anti-inflammatory activity of heparin, responsible for inhibition of adhesion of leukocytes to the activated endothelium, in conjunction with its anticoagulant activity, had been proposed previously and may, in this model, participate in the antithrombotic effect of heparin.^27^

In clinical trials, it has been observed that statins, and particularly rosuvastatin, exert a certain protective effect against development of venous thromboembolism.^28^ It has been suggested that one of the mechanisms of this effect is the anti-inflammatory role played by these medications. However, these results are considered preliminary and additional evidence is needed to justify using these drugs for this purpose.^29^^-^ 31

The results observed with these various different substances with anti-inflammatory activity in the different animal models of VT and, in the case of statins, in clinical studies, are encouraging and suggest the possibility that we are on course towards a new class of medications which, with little or no hemorrhagic effect, can be used for prophylaxis and treatment of venous thromboses with greater safety. These results also suggest that the prophylactic effect of anticoagulants used at doses lower than those for treatment of VT may be, at least in part, because of a local anti-inflammatory effect.
