# Supplementary material for: Future prospects for prophylactic and therapeutic management of venous thrombosis: antithrombotic substances with lower risk of hemorrhage?
Source: J Vasc Bras. 2019 May 28;18:e20190036. doi: 10.1590/1677-5449.190036 (PMC6634938; doi:10.1590/1677-5449.190036)
Supplement: Translation

# Translation: Perspectivas futuras na abordagem profilática e terapêutica da trombose venosa: substâncias antitrombóticas com menor risco hemorrágico?

**Authors:** Francisco Humberto de Abreu Maffei

**Affiliations:** 1 Universidade Estadual Paulista – UNESP, Faculdade de Medicina de Botucatu, Departamento de Cirurgia e Ortopedia, Botucatu, SP, Brasil.

Os anticoagulantes são medicamentos essenciais no tratamento e na profilaxia do tromboembolismo venoso desde a década de 40 do século passado, com a utilização da heparina e das anti-vitaminas K. Porém, mesmo com a introdução das heparinas de baixo peso molecular, do fondaparinux e, mais recentemente, dos anticoagulantes orais diretos, existe ainda um risco não desprezível de hemorragias significativas e mesmo letais^1^^-^ 4.

Os conhecimentos adquiridos nas últimas décadas, principalmente a partir de estudos experimentais, sugerem que o sistema imunológico e as células inflamatórias têm papel na ativação inicial e localizada da coagulação nas veias que desencadeia a trombose, principalmente nas situações em que existe diminuição ou bloqueio do fluxo sanguíneo^5^^-^ 8. Essa situação de estase ocorre no ser humano em casos de compressão venosa e de repouso no leito por doença clínica ou cirurgia, e também durante anestesia, imobilização por trauma, paralisia e viagens prolongadas^9^^-^ 11.

Estudos em modelos experimentais de trombose provocada pela diminuição ou parada de fluxo sanguíneo induzidas por estenose ou ligadura em veia cava de roedores, sugerem que, em decorrência da isquemia e da ativação das células endoteliais, haveria liberação de moléculas que atraem leucócitos e plaquetas e também exposição de moléculas de adesão dessas células ao endotélio. Foi demonstrado também que os leucócitos aderidos são principalmente monócitos que liberam fator tecidual (FT) e neutrófilos que liberam enzimas e formam as armadilhas extracelulares de neutrófilos (*neutrophil extracellular traps*, NETs), que ativam fatores de coagulação e inativam anticoagulantes naturais^12^^-^ 14. Von Brühn et al.^15^ mostraram, de maneira muito ilustrativa, por microscopia eletrônica de varredura, que a realização de estenose na veia cava de camundongos não provoca lesão morfológica do endotélio. Porém, os autores observaram que, após 1 hora, se iniciava o rolamento de leucócitos sobre o endotélio, e que, após 6 horas, a superfície endotelial estava coberta por uma camada dessas células. Mostraram também, por microscopia intravital, que os leucócitos eram principalmente monócitos e neutrófilos. Mostraram, ainda nos trombos, a presença de NETs e seu papel na formação e na progressão do trombo pela ativação do sistema intrínseco da coagulação. Para tal, utilizaram animais transgênicos com neutropenia ou com deficiência de fator XII e animais em que as NETs foram lizadas por DNase, nos quais não havia formação do trombo.

Surge então a pergunta: seria possível utilizar, na terapia da trombose venosa (TV), substâncias com ação anti-inflamatória que inibissem esses mecanismos e com menor ou nenhuma ação anticoagulante sistêmica?

Baseados nos conhecimentos acima referidos, foram utilizadas diferentes substâncias com a finalidade de inibir moléculas de atração ou adesão de células inflamatórias, como as P e as E-selectinas, ou inibir enzimas liberadas por essas células, que ativam localmente o sistema de coagulação ou agem em algum ponto dessa sequência de eventos iniciais no desenvolvimento de trombos, visando impedir sua formação ou progressão, sem interferir na coagulação sistêmica.

Inúmeros trabalhos do grupo do Dr. Wakefield, da Universidade de Michigan, mostraram a ação antitrombótica de inibidores da P-selectina, molécula de adesão de plaquetas e leucócitos, tanto em modelo de trombose induzida por ligadura de veia cava de ratos^16^^,^ 17 como em macacos, induzindo a trombose na veia cava ou em veias ilíacas, por meio de balão oclusor^18^^-^ 21.

Culmer et al.^22^, do mesmo grupo, utilizando um inibidor da E-selectina para prevenção e tratamento de trombose em modelo de estase na veia cava de camundongos, também mostraram efeito inibidor da formação e extensão de trombos de maneira similar à enoxaparina, em relação ao grupo controle, sem alteração do tempo de sangramento (TS), como ocorreu com a enoxaparina.

Em um trabalho nessa linha testando substâncias com ação anti-inflamatória na prevenção da TV, realizado no Laboratório de Purificação de Proteínas do Departamento de Bioquímica da UNIFESP (Profa. Dra. Maria Luiza Vilela Oliva), utilizamos o inibidor recombinante rBbCI^23^, cuja proteína original demonstrou ter ação inibidora de elastase, de catepsina-G^24^, enzimas pró-inflamatórias também inibidas pela heparina^25^. Além disso, o rBbCI reduziu os níveis de interleucina-8, citocina que estimula principalmente a migração de neutrófilos para o foco inflamatório^24^. No modelo de ligadura da veia cava em ratos, o rBbCI teve ação inibitória do desenvolvimento do trombo similar à da heparina, e como ela, teve ação dose dependente^26^. O rBbCI não alterou o tempo de tromboplastina parcial ativada nem o TS na cauda dos animais, que foram iguais aos dos animais controle. A ação anti-inflamatória da heparina, responsável pela inibição da adesão de leucócitos ao endotélio ativado juntamente com sua ação anticoagulante, já havia sido levantada anteriormente, podendo, nesse modelo, ter participação no efeito antitrombótico da heparina^27^.

Em ensaios clínicos, foi verificado que as estatinas, principalmente a rosuvastatina, exercem um certo efeito protetor contra o desenvolvimento do tromboembolismo venoso^28^, sendo sugerido como um dos mecanismos dessa ação o papel anti-inflamatório desses medicamentos. Entretanto, são resultados considerados preliminares, necessitando novas evidências para justificar a utilização de tais medicamentos com essa indicação^29^^-^ 31.

Os resultados verificados com essas várias substâncias com ação anti-inflamatória em diferentes modelos animais de TV e, no caso das estatinas, em estudos clínicos, são animadores e sugerem a possibilidade de estarmos no caminho de uma nova classe de medicamentos que, com pouco ou nenhum efeito hemorrágico, possam ser utilizados com mais segurança na profilaxia e no tratamento das tromboses venosas. Esses resultados sugerem também que o efeito profilático de anticoagulantes usados em doses menores do que as de tratamento da TV possa ser, pelo menos em parte, devido a uma ação anti-inflamatória local.
